# The role of cellular traction forces in deciphering nuclear mechanics

**DOI:** 10.1186/s40824-022-00289-z

**Published:** 2022-09-08

**Authors:** Rakesh Joshi, Seong-Beom Han, Won-Ki Cho, Dong-Hwee Kim

**Affiliations:** 1 KU-KIST Graduate School of Converging Science and Technology, Korea University, Seoul, South Korea; 2 Department of Biological Sciences, Korea Advanced Institute of Science and Technology (KAIST), Daejeon, 34141 South Korea; 3 Department of Integrative Energy Engineering, College of Engineering, Korea University, Seoul, South Korea

**Keywords:** Traction force microscopy, Biomaterials, Mechanobiology, Nuclear mechanics

## Abstract

Cellular forces exerted on the extracellular matrix (ECM) during adhesion and migration under physiological and pathological conditions regulate not only the overall cell morphology but also nuclear deformation. Nuclear deformation can alter gene expression, integrity of the nuclear envelope, nucleus-cytoskeletal connection, chromatin architecture, and, in some cases, DNA damage responses. Although nuclear deformation is caused by the transfer of forces from the ECM to the nucleus, the role of intracellular organelles in force transfer remains unclear and a challenging area of study. To elucidate nuclear mechanics, various factors such as appropriate biomaterial properties, processing route, cellular force measurement technique, and micromanipulation of nuclear forces must be understood. In the initial phase of this review, we focused on various engineered biomaterials (natural and synthetic extracellular matrices) and their manufacturing routes along with the properties required to mimic the tumor microenvironment. Furthermore, we discussed the principle of tools used to measure the cellular traction force generated during cell adhesion and migration, followed by recently developed techniques to gauge nuclear mechanics. In the last phase of this review, we outlined the principle of traction force microscopy (TFM), challenges in the remodeling of traction forces, microbead displacement tracking algorithm, data transformation from bead movement, and extension of 2-dimensional TFM to multiscale TFM.

## Background

The transfer of mechanical forces from the extracellular matrix (ECM) to the cell not only affects the nuclear shape and cytoskeletal reorganization but also gene expression and remodeling of nuclear chromatins [1–6]. During cellular mechanosensation, cells respond to mechanical stimuli in their environment that in turn regulates important processes in normal as well as diseased states [7–10]. Cells in the body are subjected to different mechanical stresses, such as compression (in the bones and other tissues), shear stress (by blood flow), and tensile stresses (due to muscle stretching), and can be interpreted in the context of mechanobiology [11–14]. Under physiological and pathological conditions, these mechanical forces are involved in inter- and intracellular functions [15]. Forces transferred from the cytoskeleton to the nucleus and correct positioning of the nucleus during cell migration are highly orchestrated because of the nuclear dimension and its resistance to deformation. These forces are generated by cellular organelles, such as filament polymerization, actomyosin contraction, and microtubule motors, which apply shearing, pulling, and compression forces on the nucleus; occasionally, these forces can rupture nuclear membranes [16, 17].

Nuclear forces are quantified by measuring the mechanical properties of subnuclear structures, including nuclear lamina, chromatin, nuclear matrix, and nuclear bodies [18]. To understand and measure cell and nuclear mechanics, a rheological approach that deals with how materials deform or flow in response to an applied mechanical force has been applied. The physical changes in nuclear properties, shape, and viscoelasticity are due to the combined effect of cytoskeletal forces, composition of the nuclear envelope, and chromatin structure. Recently, chromatin has emerged as a key contributor to overall nuclear stiffness in cancer cells [19].

Determining cell adhesion, migration, and invasion phenotypes of the cell, along with understanding the fundamental molecular mechanisms in the diagnosis, treatment, and drug development, depends largely upon ECM modeling and their fabrication route, techniques used, experimental design, and the errors associated with them. Numerous experimental methods, including simple washing assays, spinning discs, and flow chambers, have been used to study cell adhesion and migration. Atomic force microscopy (AFM), micropipettes, particle tracking, magnetic bead microrheology, and cellular deformation by optical force and fluidic shear stress have been applied to the single cells [20].

The aim of this review is to explore the various types of ECM used in mechanobiology and the challenges faced in achieving these approaches. Subsequently, we reviewed the various forces transferred from the ECM that are experienced by cells and the practical techniques used to measure them during cell adhesion and migration. We also reviewed the principles, advances, challenges, and various errors associated with traction force microscopy (TFM) in single-cell systems.

## The role of ECM in subcellular mechanics

### Engineered ECM

The ECM is a 3-dimensional (D) matrix network that provides structural support and stability to organs and generates and transfers signals from one part of the body to another. Treating certain diseases requires ECM remodeling as it plays a crucial role in cellular activities and can be targeted as a therapeutic strategy [21]. The signal generated from the interaction of cells with the ECM is fundamentally dependent on molecular functions and cellular phenotypes [22]. Thus, for the selection of suitable biomaterials that adequately reproduce *in vivo* tissue conditions, it is important to consider their biocompatibility to determine whether the biomaterial generates an adverse reaction in the body as well as physiological aspects of materials such as degradation kinetics and mechanical properties, e.g., elasticity, tensile strength, elongation, and hardness [23–27].

Previous studies have revealed that the mechanical properties of the ECM directly affect the regulation of cell function [28]. The available natural ECM matrices, such as collagen, chitosan, fibrin, gelatin, alginate, and hyaluronic acid, have a sophisticated system composed of water, proteins, and polysaccharides with unique topology and composition that together provide the correct chemical, biological, and mechanical environment for cells [29–31]. Primary cells and tissues are the two primary sources of natural ECM. Human and animal tissues are widely used to manufacture natural ECM products [32, 33]. The fibers and large number of pores in the ECM generate a low-concentration stiff matrix that allows the migration of cells, whereas the complex stiffness of the ECM causes stress or strain stiffening [34].

Engineered biomaterial-based ECM can induce desirable cell-specific responses to mimic tumors and their microenvironment [35]. Many researchers have used the ECM to study cell behavior during stem cell proliferation and differentiation [36, 37] and disease progression [38]. The ECM is highly heterogeneous and composed of fiber-forming and non-fiber-forming proteins, such as collagens and elastic fibers, proteoglycans, glycosaminoglycans, and soluble factors [39–41]. Organ-specific cells bind to the ECM via surface receptors to activate cell responses including growth, migration, proliferation, and differentiation [42].

While designing an ECM to mimic tumors and their microenvironment, ECM protein coating, electrospinning, hydrogels, and 3D bioprinting techniques are often applied [41]. 3D printing techniques usually use organic solvents, high temperatures, or crosslinking agents, which are harsh for the biological ECMs. Therefore, an appropriate printing material with superior biocompatibility, printability, desired degradation, and mechanical properties should be selected [43]. Natural ECM shows excellent biocompatibility but weak mechanical properties despite its crosslinking characteristics. On the other hand, the properties of synthetic materials such as polyetheretherketone (PEEK), polycaprolactone (PCL), polyethylene glycol (PEG), and polylactic acid (PLA) can be easily manipulated to suit experimental needs [44]; however, their poor biocompatibility, lack of bioactive ligands, and toxic degradation render 3D printing more complex [45].

**Fig. 1:**
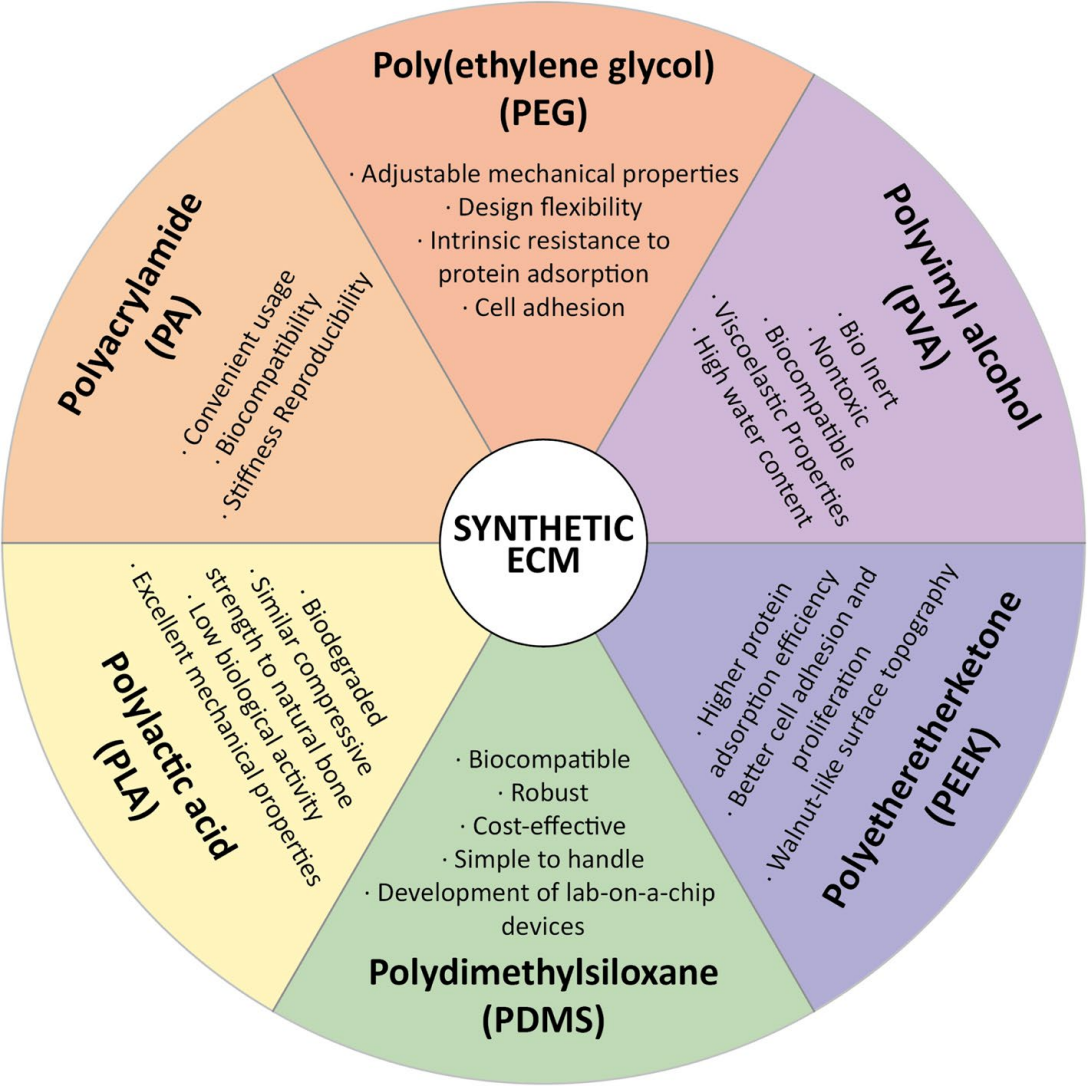
Available synthetic extracellular matrix (ECM). Various types of synthetic extracellular matrix biomaterials and their properties that mimic tumor microenvironment

Synthetic hydrogels have proved useful to culture primary cells in 2D and 3D microenvironments and to test the role of ECM in the regulation of cell function. Water-soluble polymers such as PEG, poly(2- hydroxyethyl methacrylate), and polyvinyl alcohol are synthetic crosslinkers forming elastic materials that can be used to mimic ECM mechanics with cells [46]. Various types of synthetic ECM and their properties have been extensively reported [47–51] (Fig. 1). Characteristics such as elasticity, morphology, wettability, and viscosity of the substrate and surface materials need to be considered when designing materials to study cell behavior [52]. Morphology, spreading, and cellular forces mainly depend on the elasticity of the hydrogel [53]. Reportedly, PEG hydrogel viscosity affects the morphology, migration, proliferation, etc. of human mesenchymal stem cells (hMSC), during tissue engineering and regenerative applications [54].

According to a study conducted in the late 1990s using polyacrylamide hydrogels with varying elastic moduli, cell-ECM adhesion, spreading, and cell migration over a cell-cultured substrate are greatly affected by substrate stiffness [55]. The mesh size of the hydrogel also affects its behavior. Cell migration efficiency is improved by employing hydrogels with smaller mesh sizes, owing to the increased migration speed [56]. In addition, the nanotopography of the ECM substrate is an important characteristic that affects cell behavior. Recently, to further determine how topography affects cell behavior, various geometric combinations, including vertical fillers, island/pits, pillars/wells, and groves, have been fabricated on rigid substrates using etching and cutting operations [57–59].

### Biophysical assessment of cell mechanics

**Fig. 2:**
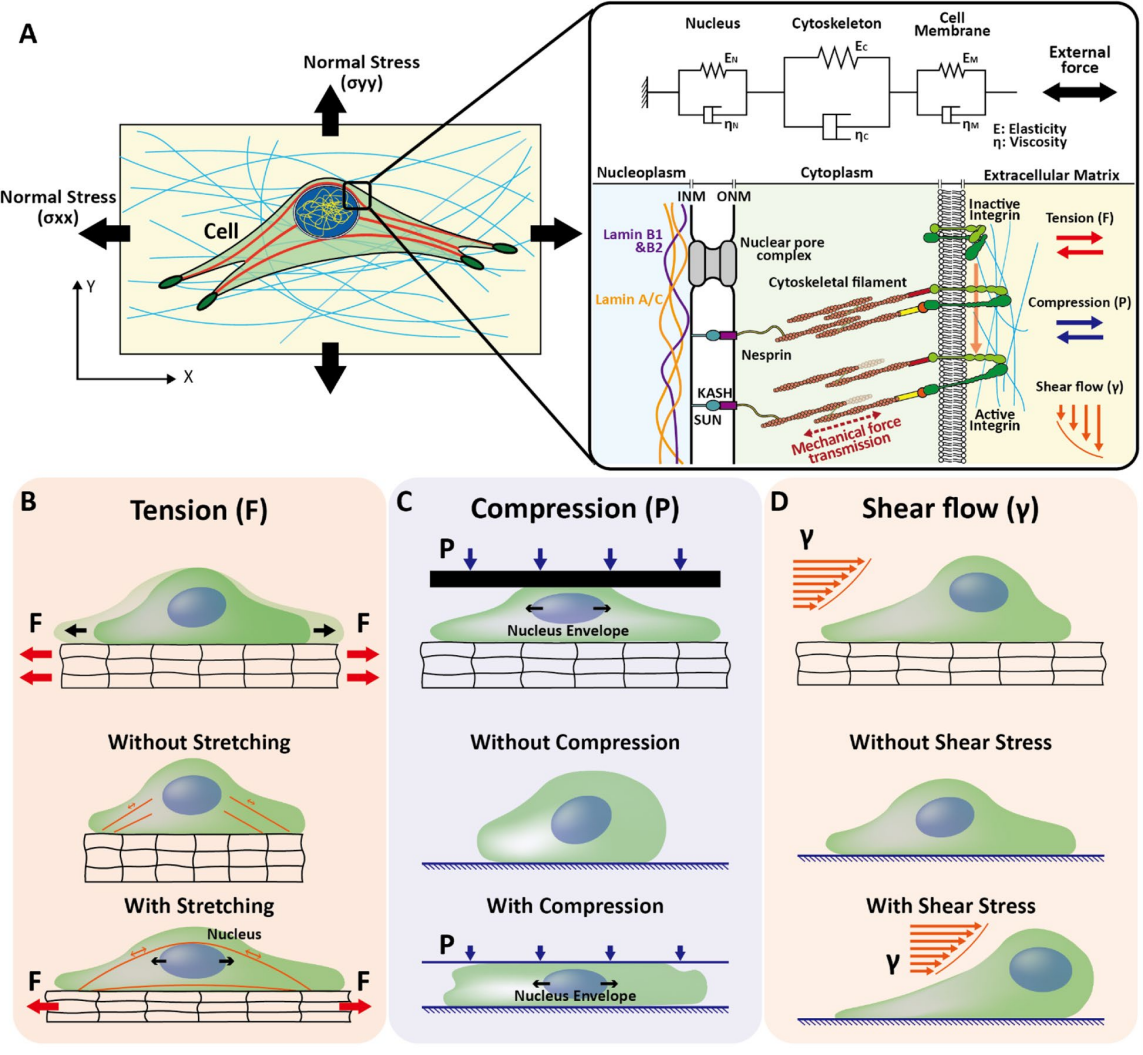
Schematic representation of forces transferred from extracellular matrix to nucleus. **A** The schematic representation of normal stress (x direction) and normal stress (y direction) on ECM. Detailed linkers of nucleoskeleton and cytoskeleton (LINC) propagates forces from extracellular matrix to nucleoplasm. Nucleoplasm domain contain nuclear envelope-associated proteins that interact with nuclear lamins. The extracellular matrix (ECM)-applied forces transfer to the nucleoplasm through cytoplasm, KASH domain protein from inner nuclear membrane (INM), and outer nuclear membrane (ONM) and enter the nucleoplasm containing SUN protein and lamins. Viscoelastic mechanical representation of Kelvin–Voigt modal (K–V) when external forces transfer from ECM to nucleus. **B** Schematic representation of the forces transferred from ECM to nucleus when ECM is subjected to tensile forces. **C** Schematic representation of forces transferred from ECM to nucleus when cell is subjected to compression forces during migration through unconfined, low confined, and strong confinement passage. **D** Schematic representation of cells on extracellular matrix during shearing flow and corresponding nucleus deformation (in deforming and peeled off) state, respectively

The common mechanical modes of forces are stretching, compressive, and shear forces as well as forces derived from surface tension continuously experienced by cells *in vivo* [60, 61]. The stress and strain experienced by cells on hydrogel surfaces are important for studying cell dynamics. The magnitude of the force experienced by the cell depends on the type of deformation experienced by the hydrogel when subjected to external stress. In general, tension, compression, torsion, and shear stresses are experienced by cells owing to the deformation of the hydrogel (ECM) during pulling, pushing, twisting, and shearing. There are two types of strain deformation based on different loading modes and the amount of force produced: plastic and elastic. Elastic deformation is recoverable compared with plastic deformation, and these deformations of the hydrogel depend on the characteristics (stiffness, viscosity, etc.) and properties (biocompatibility, toxicity, etc.) of the ECM (Fig. 2) [62]. In this section, we summarize various methods to study the mechanical properties of cells embedded in deformable hydrogels.

The micropipette aspiration (MA) system uses microinjection to create suction pressure in a capillary, where the cell is directly contacted by a microneedle or a microcapillary under a microscope. MA measures cellular viscosity by measuring the negative pressure needed to maintain the suction length and is capable of measuring forces in the range of 10 pN–1 nN [63]. This technique is useful for measuring changes in the cell shape and viscosity of single cells [64]. Magnetic tweezer-based measurement involves non-invasive manipulation of magnetic particles, e.g., a superparamagnetic Fe_2_O_3_ nanoparticle-embedded polymer matrix attached to a biological entity via an external magnetic field. The magnetic particle positions were tracked in a magnetic field using a microscope. Magnetic tweezers can measure forces of up to 100 pN [65]. The optical tweezer method relies on the concept that light changes its path when it passes through two different mediums with different refractive indices. During the experimentation process, the small particles are attached to the cell membrane and pulled away from the cell, creating a tethered membrane; light passing through a pulled cell changes direction (i.e., refraction) and alters momentum [66]. The intercellular forces measured with optical tweezers have been reported to be in the range of 50–300 pN [67]. Owing to their ability to control the cellular microenvironment, microfluidic and organ-on-a-chip systems have become powerful tools for cell biologists. For instance, a polydimethylsiloxane (PDMS)-based transparent microfluidic compression device was designed by employing a self-adhesive to evaluate the cellular deformation of cells using optical microscopy [68], which was applied to control the magnitude of the applied pressure to inflict compressive strain and evaluate Young’s modulus in the range of 3.5–4.2 kPa.

Among various cellular force measurement techniques, AFM has been the most conventionally utilized technique because it can characterize mechanical properties, including the elasticity and viscoelasticity of multiple cell types [69–73]. The accuracy of AFM results depends on the type of tip; generally, AFM using a spherical probe is utilized to measure whole-cell properties, owing to the low elastic modulus of cells. AFM has also been used to measure cell-substrate adhesion by modifying the AFM cantilever. However, this technique is tedious and sometimes very difficult to estimate whether the cell-substrate interaction is stronger than that of the cell cantilever.

Recently, fluidic force microscopy was applied to study single-cell mechanics and cell-to-material adhesion using a fluidic force fluid tip positioned with an AFM tip holder [61]. Subsequently, negative pressure was applied to attach the cell to the cantilever, and by applying force to the cell on the cantilever, the force was sensed using a standard feedback sensor, i.e., an AFM laser detection system. Another group also combined the AFM with optical tracking to study viscosity and elastic components using 3T3 fibroblast cells [74]. For this experiment, the authors used micrometer beads attached to an AFM tip and indented it on the cell surface ranging from 0.2–1.2 μm in depth. The cellular response corresponding to a lower force range (10–30 pN), measured using the optical track technique, showed a reversible elastic response owing to the conversion of the viscous effect into the apparent elastic modulus.

Researchers have also used the finite element modeling (FEM) to estimate the elasticity of a cell by comparing it with the results obtained using AFM, comparing the force-indentation depth response of the cell, and predicting the intercellular force transduction and distribution [75]. Nanoindentation is another AFM method. While the modulus map obtained from conventional AFM is higher than that obtained from nanoindentation [76], the latter enables depth sensing that recodes load (P), depth (d), and time (t). This apparatus allows for controlled indentation force and displacement and is particularly useful for live cells and subcellular components [69].

In addition to the abovementioned techniques, micropillar-based devices and TFM-based techniques have been utilized. In micropillar-based techniques, different grades (e.g., sparse and dense zones) of micropillar-coated substrates have been developed [77], in which cells in the sparse zone are more aligned along the direction of the micropillar spacing gradient. In another study, a poly(lactide-co-glycolide) (PLGA) polymeric micropillar array with an appropriate dimension was used to study the changes in the shape of mesenchymal stem cells over time [78]. In a recent study, magneto-active micropillar arrays (poly(n-butylacrylate)) were fabricated by mixing prepolymers with magnetic iron oxide nanoparticles [79], where the pillar surface was designed in such a way that its top faces attracted the cells, and the other side of the pillar wall along with the area between the pillars was covered with a cell-repelling hydrogel layer. Accordingly, pillar deflection owing to magnetic stimulation generates traction forces on the adherent cells.

Another technique for measuring the cell-generated force exerted by a cell lying on a soft surface is the 3D TFM, an important force measurement technique that works on the knowledge of the displacement-force, elastic modulus, and mechanical properties of the substrate [80]. These cell-generated traction forces deform the soft substrate and lead to the formation of a deformation field. In the TFM experiments, cells were cultivated on fluorescent microsphere bead-embedded ECM-mimicking substrates to investigate the cell-generating traction forces by measuring the change in bead displacement (refer to the details of TFM in Sect. 3).

Recently, image-based mechanical analysis techniques, such as coherent optical methods of stress analysis and electronic speckle pattern interferometry (ESPI) have been used by many researchers [81]. According to Harris and colleagues, when cells are cultured on a soft elastic substrate like a thin sheet of silicon, they exert a traction force responsible for elastic distortion and wrinkling of the substrate [82]. Individual wrinkles change during cell migration owing to changes in cellular forces and can be detected using a machine learning approach [83]. They constructed a machine learning system by obtaining TFM images and comparing them with cell-generated wrinkle images using a convolution neural network. They reported that the trained model was able to predict the cellular force using microscopy images alone.

### Deciphering nuclear mechanics

Understanding nuclear mechanics is crucial for many diseases such as cancer and premature aging syndrome [19]. During cell division, migration, and differentiation in eukaryotic cells, the nucleus is actively positioned at specific locations within the cytoplasm [84]. During cell migration in confinement, the nucleus deforms when the cell passes through a small constriction. The estimation of the force required to deform the nucleus is an important question to be addressed [85]. The direction of cell migration is specified by the position of the nucleus within the cell, which is usually positioned towards the rear. Therefore, it is also important to comprehensively understand the complex roles of focal adhesion, the cytoskeleton, and their connection to the nucleus for proper cell migration. During cell migration *in vivo*, cells need to pass through the 3D ECM system, where all intracellular components, including the cytoplasm, plasma membrane, and intracellular structures, deform the nucleus, which is a gigantic organelle generally larger than the pore size.

The transfer of force from the ECM through the cytoskeleton can deform the nucleus. The external forces applied to the ECM were transmitted to the nucleus via the cytoplasm and analyzed mechanically using the Kelvin–Voigt model (K–V). In the K–V mechanical model, the cell membrane, cytoplasm, and nucleus are connected in series and each represents a spring (elasticity) and damper (viscosity) (Fig. 2A) [86]. The viscoelastic and elastic nature of the nucleus protects genetic material from applied forces [87] and improves the flexibility of cell motility through the constricted region [88]. The amount of force transferred from the ECM to the nucleus (causing nuclear deformation) depends on the magnitude of elasticity and viscosity of the cell and ECM. Cellular and nuclear elasticity and viscosity play important roles in cellular mechanics [86]. The large magnitude of these forces can generate large deformations in the nucleus and cause uncontrolled nucleus–cytoplasm content exchange, loss of nuclear envelope content, DNA damage, and sometimes cell death [88].

The ECM microenvironment propagates forces into the nucleus through the LINC (linker of nucleoskeleton and cytoskeleton) complex, which connects the nucleus to cytoskeleton [89]. Unwanted extracellular forces and defective forces disrupt the LINC complex, which later disrupts micro-sensitive gene expression. This transfer of force from the cytoskeleton regulates nuclear shape and orientation. Recent studies have revealed that the accumulation of aberrant actin fibers around the nucleus could induce actin-dependent nuclear deformation, causing nuclear tension and leading to herniation, blebbing, and sometimes nuclear envelope rupture [90]. Actin fibers play an important role not only in coordinating intricate architecture but also in protecting genetic information [91]. Because the nucleus is generally considered to be 5–10 times stiffer than the cytoskeleton, the structural change in the nucleus envelope and chromatin due to external mechanical loading depends on the stiffness of the nucleus and surrounding cytoskeleton [92]. In addition to structural changes in the nuclear envelope, chromatin- and cytoskeleton-mediated changes can also be induced by tailoring the mechanical properties of the nuclear envelope.

Recently, the relationship between the expression of nuclear lamin A scaled with tissue stiffness and matrix rigidity-dependent cell differentiation showed that increasing substrate stiffness increases lamin A and C levels in MSCs and myosin II contractility [93]. Moreover, alterations in nuclear morphology, lamin A/C expression, and epigenetic modifications were induced by changes in the tensile force arising from the cultivation of fibroblast cells on fibronectin-coated micropatterns [94].

The structure of the nucleus and its mechanics change in various human diseases, and there is a functional relationship between cellular dysfunction and nuclear morphology. Cell nuclei are the largest organelles and are subjected to continuous mechanical loading, such as tensile, shear, and compressive stress, which regulate signaling pathways, cytoskeleton remodeling, and musculoskeletal function within multicellular organisms [95]. To understand the external force transmission mechanism from the ECM, understanding the connection between each component from the nucleus to the ECM is an important aspect to study. Transmembrane protein integrin is a bi-directional signaling receptor that forms a link between the interior of the cell and its extracellular environment as well as forms an adhesion site (by anchoring the cell with ECM) [96, 97]. The other side of integrin is attached to the cytoskeleton tensile member (actin filaments) through the focal adhesion complex. The actin filament structure inside the cytoplasm holds the nucleus and is responsible for maintaining the shape of the cell (when cells are spread over the ECM). Subsequently, these forces from the cytoskeleton propagate to the nuclear surface from integrin receptors with the help of F-actin. External forces are transferred from the ECM to the plasma membrane and stimulate the force-sensitive opening of ion channels. The transfer of forces from the cell surface to the nucleus by applying tensile forces to the endothelial cell surface was confirmed by observing the nuclear envelope distortion [98]. According to a study conducted on MSCs, when external tensile forces are applied to the ECM, changes in chromatin organization condensation stiffen the MSC nucleus and reduce its deformability against externally applied tensile stretch [99]. We previously reported that 1 h of uniaxial cyclic stretch alters the 3D shape of the nucleus (Fig. 2B) [100], whereas nuclear flattening stretches the pores of the nucleus and decreases the mechanical passages for molecular transport, resulting in the accumulation of the mechanosensitive transcription factor YAP in the nucleus [101].

During cancer metastasis, cells migrate through narrow constricted channels, which causes nuclear distortion. The compression forces transferred from the ECM significantly decrease nuclear height, and increase nucleus area. These dimensional changes due to external ECM forces produce a volumetric strain in the nucleus. (Fig. 2C).

In the case of ECM subjected to shearing force, cells in the body (generated from blood flow or interstitial flow) sense shear stress via a series of receptors and play a critical role in modulating cell function [102]. These receptors transmit mechanical signals through mechanosensitive pathways and regulate the cell morphology. Under shear stress, endothelial cells are elongated, and their stress fibers are aligned along the direction of fluid flow. In an experimental study on the measurement of shear deformation of fibroblast cells using an on-chip TFM, various stages of deformation were studied. In the first stage of deformation, the flowing fluid applied stress over the top layer of the cell surface (plasma membrane), spread the cell, and was responsible for the cell and nucleus deformation. In the second stage of deformation, these fluid dynamic forces provided rotation from the leading side of the cells and started peeling off the rear side of the cell (Fig. 2D). In the third stage of deformation, cells detached from the ECM. These fluid shear stress (FSS) (unit N/m^2^ or dynes/cm^2^) is expressed as the product of the shear rate and viscosity of the fluid [103]. Cells in laminar fluid shear are responsible for cell elongation, suppression of proliferation, cytoskeleton organization, and redistribution of focal adhesions. The nucleus inside the cell experiences forces transferred from the ECM during external loading conditions, causing deformation of the nucleus [104, 105]. In an experimental study in which Madin-Darby canine kidney cells were subjected to FSS (1.1 dynes/cm^2^) in a microfluid channel, an approximately 50% reduction of nuclear area was reported [106].

The transmission of forces from the ECM to the nucleus depends on the magnitude of forces applied to the ECM. The viscosity and elasticity of the ECM and cytoplasm absorb the transfer of forces from the ECM to the nucleus. In an experimental study conducted by researchers to characterize the anisotropic deformation of NIH 3T3 cell nuclei in response to extracellular forces by quantitative measurement of nuclear strains, it was reported that nuclear anisotropy properties may provide a direct mechanism to transfer force via the ECM to the genome [16]. The deformation mode depends on the type of loading experienced by the cell. In one study, the authors measured the extreme deformation of the nucleus during cell migration in the confinement space (Fig. 2C). When cells pass through this confinement region, the nucleus has to deform, and the deformed shape of the nucleus can be easily explained using experimental images. The degree of deformation in the nucleus inversely depends on the confinement size. However, direct experimental measurement of the minimum forces required to deform the nucleus is extremely difficult [85]. The use of hybrid microfluidic and TFM techniques can be a better solution for measuring the forces that cause nuclear deformation.

The externally applied forces induce a response within the cell to the nucleus and are responsible for the change in cell behavior and cell fate [107]. In an experimental study on mammary carcinoma cells under compression stress, compression stress accumulation during tumor growth prevented the migration of cancer cells by enhancing cell-substrate adhesion [108]. When cells undergo sharing forces by applying fluid flow, they impinge on human MSCs and regulate cell fate by regulating stem cell differentiation via the contractility of the stem cells [109]. Neural stem and progenitor cells subjected to 10% static equiaxial stretch specifically affect differentiation, and this effect is dependent on ECM integrin linkage [110]. A study on embryonic stem cells (ESC) investigated the relationship between shape mechanics and cell fate. They found that shape change is regulated by a catenin-mediated decrease in RhoA activity, which decreases plasma membrane tension and results in early differentiation defects in ESC [111].

**Fig. 3:**
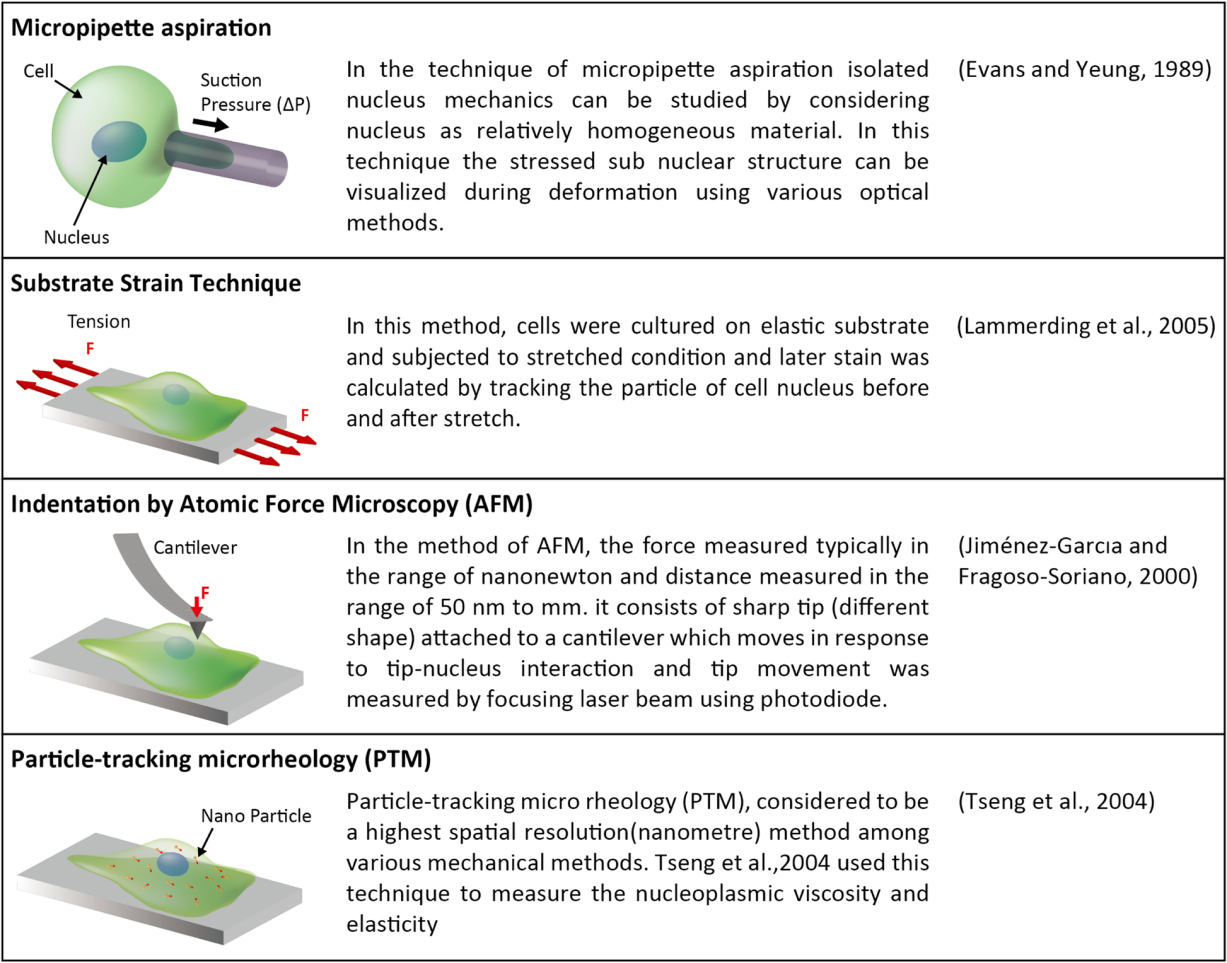
Conventional techniques to study cell nucleus mechanics. Brief descriptions are listed for micropipette aspiration, substrate strain technique, indentation atomic force microscopy (AFM), and particle-tracking microrheology (PTM)

Nuclear mechanics can be studied either within cells or by isolating the nucleus from cells. Nucleus isolation studies allow stress to be applied directly to the nucleus rather than to the cytoplasm [112]. Experimental methods for measuring nuclear properties by isolating cells increase uncertainty regarding the effects of the chemical environment. MA, AFM, substrate strain testing, and particle tracking microrheology techniques have also been used by many researchers (Fig. 3) [113–116].

Therefore, accumulating evidence supports that externally applied tensile forces propagate along the cytoplasmic filament and are transmitted to the nuclear lamina across the nucleus-cytoskeletal connection, ultimately regulating the nuclear forces.

## Effects of traction forces on cell adhesion and migration

### Engineered systems to measure forces during cell motility

The ability of cells to physio-chemically adhere to the ECM is known as cell adhesion that plays a fundamental role in the maintenance and development of tissues [95]. The mechanical force experienced by cell–cell and cell-ECM junctions stretches, aligns, bends, and repositions tissues and regulates cell functions, including signaling, differentiation, proliferation, pattern formation, and migration [117]. Changes in cell adhesion are responsible for a wide range of diseases, including osteoporosis [118], atherosclerosis [119], arthritis [120], and cancer [121]. For example, adhesion between blood cells and platelets leads to heart failure owing to the formation of blood clots, restricting the blood supply to the heart muscles [122].

**Fig. 4:**
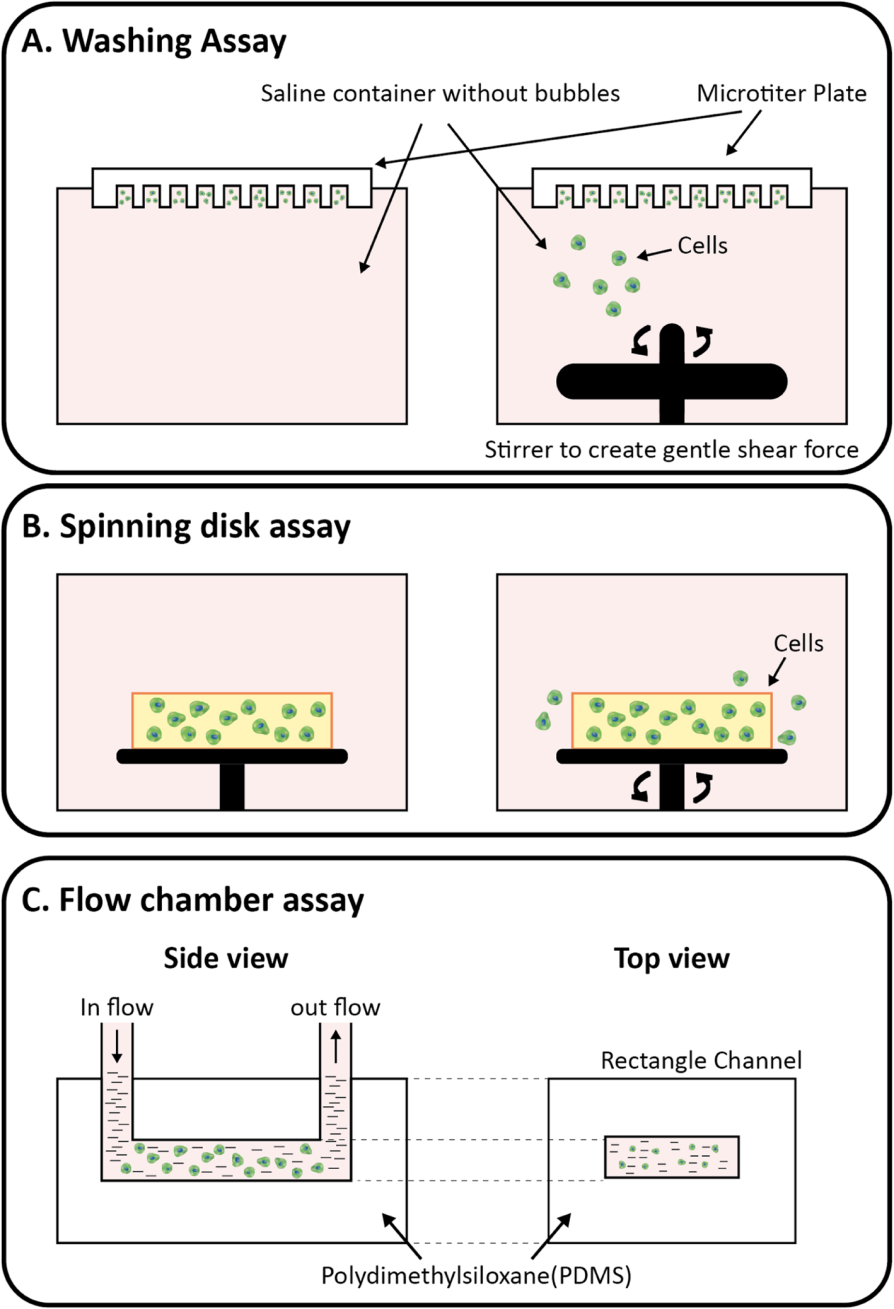
Techniques to study cellular adhesion of multiple cells. **A** Schematic of the simple washing technique: (left) cell adhesion state without any shear force, (right) cell under gentle shear stress owing to the stirrer. **B** Schematic of the spinning disk technique: (left) cells in culture dish without the application of external centrifugal forces (reference state), (right) cells moving out from culture dish owing to the application of applied centrifugal force by spinning disk. **C** Schematic of the flow chamber technique: adhering cells were measured at different flow speeds using polydimethylsiloxane (PDMS) rectangular microchannel device

Mechanotransduction, the conversion of mechanical signals into biochemical responses, requires integrins, myosin motors, cytoskeletal filaments, nuclei, and ECM to generate forces during cell migration [123, 124]. Cells continuously apply various forces such as pushing, pulling, and shearing on the ECM, which determine cell migration and tissue morphogenesis. These forces not only trigger signaling pathways but also guide mechanical and structural events under both physiological and pathological conditions [125]. According to recent studies on the force-motion relationship [126], there are four different types of forces: internal forces, inertial forces, forces from fluid, and forces from the ECM. Many researchers have reported techniques for studying cell adhesion such as 1) simple washing, 2) spinning disks, and 3) flow chambers (Fig. 4).

A simple washing technique was used to measure weak cell adhesion, whereas a minimal shear force was applied to the fluid to remove nonadherent cells. This technique is easy to perform by applying a defined amount of shear force to the fluid to measure cell adhesion on different ECM substrates [127]. The cells were cultured on different ECM substrates using the spinning disk technique for cell adhesion measurements. The culture dish was then mounted over a spinning disk and rotated at a defined revolution per minute (RPM), and cell adhesion with different substrates was quantified before and after rotation using optical microscopy [128]. In another study, PDMS was used to construct a flow channel device with dimensions of 20 × 1000 × 160 mm, and inlet and outlet tubing were attached to the two extreme ends of the rectangular channel [129]. Cells were then seeded inside the chamber and exposed to different experimental conditions: no flow for 60 min and 330 mL/s for 30 min, and the cells were counted under different conditions using an optical microscope.

The abovementioned techniques cannot measure single-cell specific adhesion, and quantification of the exact shear stress force is difficult; therefore, their applications are limited only to weakly adherent cells. Single-cell force spectroscopy and biomembrane force probes have been developed to overcome these issues.

An AFM probe is used to physically detach the individual cell from an open medium, and the detachment force is quantified by measuring the deflection of the probe used to detach the cell divided by the cell area [130]; in single-cell force spectroscopy, image-based cell adhesion is quantified at the single-cell level. This technique uses a microscope to capture the cell and its structure, mechanics of isolated biomolecules, subcellular cytoskeleton, and components of the nucleus in assistance with nano- and micro-manipulators to determine the mechanical properties of various cell types [131–133]. The biomembrane force probe is a sensitive and feasible experimental technique that allows quantification of single-molecule bonds and is used to measure force in the range of 0.1 pN to 1 nN with a loading rate of 1–106 pN/s [134].

Other experimental techniques, such as micropillar- and embedded sensor-based techniques, have been used by many researchers [60, 135]. Of these techniques, however, TFM remains the most widely used technique for measuring cell traction forces for its versatile applications [136, 137].

### Working principle of traction force microscopy

Adherent cells must attach to a suitable substrate or ECM for their growth and survival. During cell adhesion, cells apply traction forces generated by actomyosin contractility to the ECM owing to the mechanical balance between internal forces and adhesion structures, which is necessary for a variety of biological processes [138–140]. TFM is a well-established technique used by biophysicists to measure the traction force generated by adherent cells in the ECM microenvironment [141]. In this technique, we directly measured the spatially resolved interfacial force of a cell cultured on a soft gel-like ECM by quantifying and analyzing soft gel deformation.

**Fig. 5:**
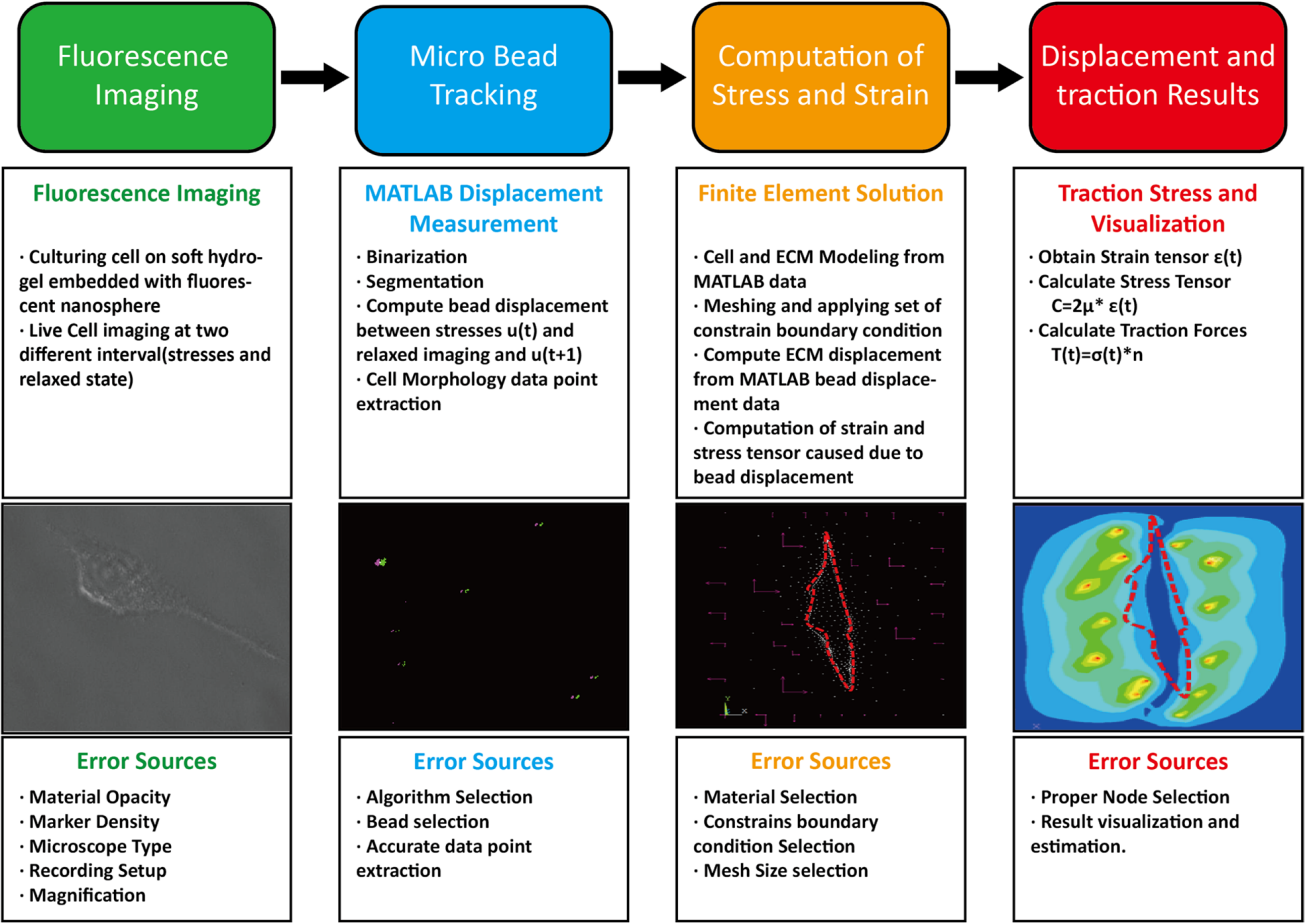
Workflow and error sources associated with the implementation of TFM. In fluorescence imaging step, microscopic images of cells were taken in after (relaxed) and before (stresses) states using trypsin. In microbead tracking, the difference in microbead positions in stressed and relaxed states was measured by employing image processing using MATLAB algorithm. The difference in microbead coordinates in stressed and relaxed images are marked with green and pink color, respectively. The microbead displacement data from MATLAB were used to compute stresses and strain during computational modeling. The cell morphology (orange) and nodes (white) along with constraint boundary condition are marked with pink arrows. The traction stress due to bead displacement and constraint boundary condition are marked with red and yellow contours in the result step

Harris et al. [82] first reported the experimental TFM concept. They demonstrated the wrinkling and elastic deformation of a thin polymeric silicon substrate when cells migrated on it. The conversion of the wrinkle pattern into traction force is not simple; additionally, this method is considered qualitative and is not currently used. Recent TFMs use optical microscopy to capture cell images, followed by image processing and force modeling. Nonetheless, the TFM is used to measure the forces exerted by cells on a soft deformable ECM. To quantify cell-generated forces, tiny fluorescent beads are embedded inside the soft ECM substrate. When a cell exerts force on the ECM, these beads deform and act as displacement markers. The positions of these markers were recorded using a microscope during cell attachment and detachment [142]. The differential deformation of these tiny fluorescent beads in the two states provides the displacement field determined using a tracking algorithm such as particle image velocimetry (PIV), particle tracking technique, image J plugin, and MATLAB combined software package. Later, these displacement data were used to model the traction stresses and strain experienced by the ECM owing to cellular force by applying a set of mechanical property data and constraint boundary conditions [143] (Fig. 5). Despite their advantages, TFM techniques are affected by many factors, such as the selection of fluorescent microbead size and type, insertion of beads into the hydrogel, volume of microbeads in the hydrogel, and quality of microscopy images [143]. As these are directly related to the TFM results, it is important to determine the sources of errors associated with the different steps of the TFM (Fig. 5).

Recently, many fundamental and technological advancements in TFM have been made to control biochemical responses and mechanotransduction at the cell–matrix interface as well as the mechanobiology behind the results. Therefore, a new artificial intelligence algorithm using machine learning to calculate the cellular force distribution from microscopy images has also been utilized [83]. In the first phase of their study, cells were cultured on an elastic substrate, and the cellular traction corresponding to the substrate wrinkle was measured, followed by the measurement of the traction force using TFM. Subsequently, the wrinkle position was captured using a microscope, and the machine learning algorithm was trained using a set of two images: extracted wrinkle images and raw microscopy images as training data for the generative adversarial network. The network was subsequently used to predict the cellular force from the input images after sufficient training. Moreover, confocal TFM has been developed [144], which involves the electrohydrodynamic nano-drip printing of quantum dots over spin-coated highly deformable silicone in the form of an array. As adherent cells generated forces and deformed the fibronectin-coated substrate, causing distortion of the nano-printed quantum dots, changes in the microscopic image of the deformable substance before and after deformation were measured using image processing.

Traction forces were remodeled using a standard TFM process. Another research group developed a high-resolution TFM technique based on an optical flow-tracking algorithm that directly measured bead deformation with an improved TFM accuracy of > 35% [145]. Two-dimensional total internal reflection fluorescence structured illumination microscopy (TIRF-SIM) and 2D TIRF-SIM-TFM were also developed by combining the TFM with a fast, super-resolution high numerical aperture [146]. They used a particle imaging velocimetry (PIV)-based simulation technique combined with live-cell experiments. The combination of magnetic tweezers with TFM further improved the TFM accuracy [147]. They used a magnetic tweezer to apply controlled force or displacement to a fibronectin-coated superparamagnetic bead attached to a collagen gel containing red fluorescent microspheres. Time-lapse imaging was used to capture the displacement of magnetic beads and microspheres. Finally, they illustrated how the control-mode bead on the hydrogel would be useful for calculating the Young’s modulus of the substrate. These approaches demonstrated that the TFM could measure the force experienced by cancer cells [148], where they used a nanometer-scale resolution for particle marker displacement using holographic tracking microscopy.

### TFM modeling and analysis

Accurate geometric modeling of cell morphology is a key step in TFM that involves (1) creation of a basic object from point lines and curves, (2) transformation (scaling, rotation, and translation), and (3) integration of elements to create the final model. Removing unwanted edges, corners, curvatures, and unwanted bumps and smoothening the model during the computer-aided design (CAD) process are crucial and directly affect the accuracy of the TFM [149]. Surface modeling, e.g., analytical surface and free-form, curved and sculptured surface, and solid modeling, e.g., constructive solid geometry, boundary representation, feature-based modeling, and parametric modeling, are the available techniques for geometric modeling, and the selection of the appropriate modeling techniques depends on the complexity of the model [150].

**Fig. 6:**
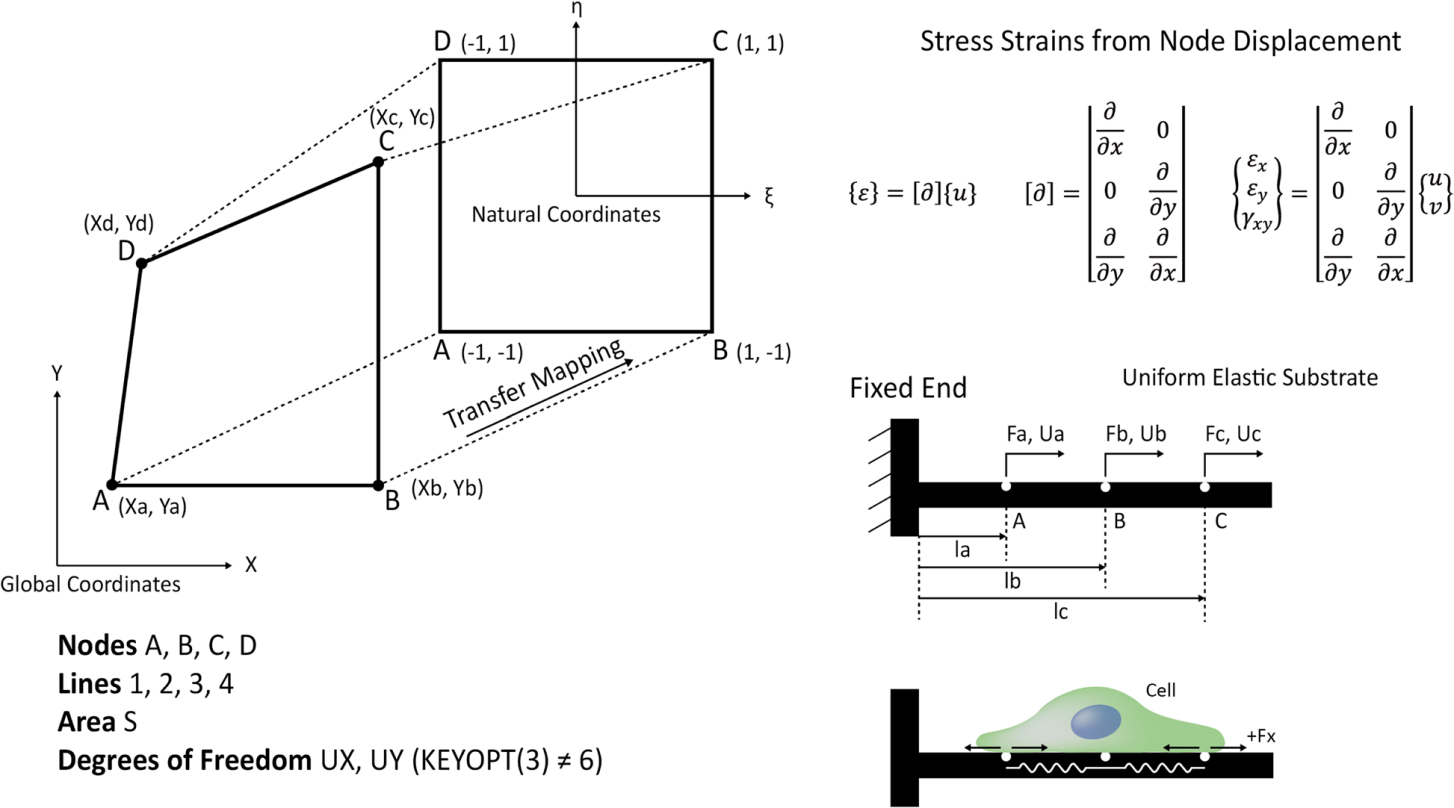
Transfer mapping from global coordinates to natural coordinates. Representation of transfer mapping of coordinates from global (x and y dimension) coordinates of nodes (E, F, G, H) to natural coordinates (A, B, C, D) using shape factor (η,ε) for finite element modeling and to calculate traction stress and strain from node displacement. The schematic representation of forces transferred from cell to the ECM (lower right)

Processing experimental data and extracting information from the TFM depends largely on the selection of an appropriate computational technique (Fig. 6). Recently, Fourier transform traction cytometry, the FEM, TFM in image J, TFM in MATLAB, and other software tools have been reported to be used [151]. The transfer mapping of microscopy image data points to natural coordinate data points for computing requires a set of conversion algorithms to convert MATLAB image data into computational modeling. In addition, various computation techniques such as the FEM, finite difference method (FDM), and finite volume method (FVM) are used to solve the computational modeling problem. Among the aforementioned computation techniques, FEM methods are widely used to solve the TFM problems. The FEM is a systematic technique that specifically solves partial differential equations for continuum elastic theory problems using a set of boundary conditions. This approach subdivides the larger model into a smaller form of a finite element, and these elements are compiled into a larger system using integration with a set of boundary conditions and constraints. Thus, the accuracy of the results depends on the selection of the element shape.

The FDM is the most direct approach for discretizing partial differential equations and is a highly efficient solution, especially for a regular grid. However, this method is not recommended for complex CAD geometries. The fundamental conversation properties of the FVM make it preferable to FDM and FEM. The FVM is a two-step process that transforms a set of complex partial differential equations into a linear algebraic equation, which affects the output results. The selection of an appropriate solver model depends not only on the level of complexity but also on the computation time required to solve the problem.

### Multiscale TFM

Most of the literature related to 2D TFM reports that cells exert only shear (parallel to the plane of the substrate) and normal traction forces (perpendicular to the substrate). How normal and shear stresses are integrated and how the magnitude of the net force is transferred to the focal adhesion and cytoskeleton are the key questions that motivate researchers to consider 3D TFM techniques [152, 153]. The recently developed 2.5D TFM over a 2D substrate precisely measured the nature of the cellular force experienced by ECM using 3D super-resolution imaging [154], which could measure multi-dimensional traction stress associated with focal adhesion and the actin cytoskeleton. In 3D TFM, by combining 3D-SIM and TFM [155], 3D-SIM was used to capture fast and spatial-resolution 3D images with minimal photobleaching and phototoxicity. Subsequently, the reference fluorescent bead embedded within the top surface of the hydrogel was captured, and the 3D traction stresses were calculated using FEM modeling. A novel technique for computing 3D displacements and traction stresses, both in-plane and out-of-plane, using epifluorescence microscopy was also studied [155]. After microscopy imaging, topology-based single-particle tracking was used to reconstruct 3D motion fields from the single-particle layer images.

The transformation of 2D TFM to 3D TFM and further multiscaling of TFMs require a complex algorithm that can guarantee critical accuracy. For instance, to determine the forces exerted by cells in multiscale TFM, researchers designed a 4D TFM technique [156], where spatial 3D system was combined with temporal 1D incorporating an ‘all in one’ toolbox to perform the necessary computation steps required for TFM. Using this technique, raw input images can be computed using a graphical user interface. The built-in free-form deformation algorithm and physical-based nonlinear inverse method render this technique suitable for computing cellular traction force in 2D, 3D, and time-lapse 4D in single- and multiple-cell systems [157–160].

## Conclusion and perspectives

Previous studies on cells and their migration have answered many but not all questions regarding the behavior of migrating cells. Cells experience mechanical stimuli from their microenvironment, which might induce growth, motion, and differentiation; this is generally termed as mechanosensation. Forces from the ECM microenvironment are transferred to the nucleus through the cytoskeleton and affect the DNA and activity of other organelles. The amount of force transferred from the ECM to the nucleus is not only related to cell morphology but also depends on ECM properties such as viscosity, stiffness, and pore size. The selection and fabrication of synthetic ECM materials with appropriate properties remain challenging. Literature from the past 10 years has revealed that there are many tools and techniques available for understanding and studying cell mechanical properties. However, the mechanism of action of these forces, particularly nuclear mechanosensation, remains unknown.

For nuclear mechanosensing mechanisms, the visualization and estimation of any changes in the cell nucleus owing to external forces have become an active area of research for biologists, chemists, and physicists; these changes also play an important role in cancer progression. Different TFM methods and modified TFM techniques have been used to analyze the force experienced by cells in the microenvironment. The integration of computational tools, such as FEM modeling, machine learning, microscopic imaging, and image analysis, has resulted in advanced techniques for studying the cellular forces generated by the external environment and estimating the nuclear forces transferred from the cytoskeleton.

The implementation of TFM in cell migration and adhesion becomes more complex when TFM is calculated in 3D compared to that in 2D. In the modeling of 3D cell morphology, the tracking of beads in one plane also remains challenging. Although the 3D traction technique is conceptually similar to 2D microscopy, the implementation of this process, selection of appropriate biomaterials, and precise spatiotemporal monitoring of bead movements in a 3D matrix complicate this process. Therefore, the standardization of bead-tracking algorithms to estimate bead displacement directly from microscopic image data can be a future research direction for the advancement of multi-dimensional and multiscale TFM applications.

## Data Availability

Not applicable.

## Abbreviations

ECM: Extracellular matrix
TFM: Traction force microscopy
PEEK: Polyetheretherketone
PCL: Polycaprolactone
PEG: Polyethylene glycol
PLA: Polylactic acid
hMSC: Human mesenchymal stem cells
MA: Micropipette aspiration
PDMS: Polydimethylsiloxane
AFM: Atomic force microscopy
FEM: Finite element modeling
PLGA: Poly(lactide-co-glycolide)
MSCs: Mesenchymal stem cells
ESPI: Electronic speckle pattern interferometry
ESCRT: Endosomal sorting complexes required for transport
RPM: Revolution per minute
PIV: Particle image velocimetry
TIRF-SIM: Total internal reflection fluorescence structured illumination microscopy
CAD: Computer-aided design
FDM: Finite difference method
FVM: Finite volume method
ESC: Embryonic stem cells
